# Effects of Calcination Holding Time on Properties of Wide Band Gap Willemite Semiconductor Nanoparticles by the Polymer Thermal Treatment Method

**DOI:** 10.3390/molecules23040873

**Published:** 2018-04-11

**Authors:** Ibrahim Mustapha Alibe, Khamirul Amin Matori, Hj Abdul Aziz Sidek, Yazid Yaakob, Umer Rashid, Ali Mustapha Alibe, Mohd Hafiz Mohd Zaid, Mohammad Zulhasif Ahmad Khiri

**Affiliations:** 1 Material Synthesis and Characterization Laboratory (MSCL), Institute of Advanced Technology (ITMA), Universiti Putra Malaysia, Serdang 43400 UPM, Selangor, Malaysia; sidek@upm.edu.my; 2 National Research Institute for Chemical Technology, Zaria, 810106 Kaduna State, Nigeria; 3 Department of Physics, Faculty of Science, Universiti Putra Malaysia, Serdang 43400 UPM, Selangor, Malaysia; yazidakob@upm.edu.my (Y.Y.); mhmzaid@gmail.com (M.H.M.Z.); mzulhasif@gmail.com (M.Z.A.K.); 4 Material Processing and Technology Laboratory (MPTL), Institute of Advanced Technology (ITMA), Universiti Putra Malaysia, Serdang 43400 UPM, Selangor, Malaysia; dr.umer.rashid@gmail.com; 5 Mechanical Engineering Department, Federal Polytechnic, Damaturu, 620221 Yobe State, Nigeria; alibebenisheikh@yahoo.com

**Keywords:** polyvinylpyrrolidone, willemite, nanoparticles, calcination, optical properties, band gap

## Abstract

Willemite is a wide band gap semiconductor used in modern day technology for optoelectronics application. In this study, a new simple technique with less energy consumption is proposed. Willemite nanoparticles (NPs) were produced via a water–based solution consisting of a metallic precursor, polyvinylpyrrolidone (PVP), and underwent a calcination process at 900 °C for several holding times between 1–4 h. The FT–IR and Raman spectra indicated the presence of metal oxide bands as well as the effective removal of PVP. The degree of the crystallization and formation of the NPs were determined by XRD. The mean crystallite size of the NPs was between 18.23–27.40 nm. The morphology, particle shape and size distribution were viewed with HR-TEM and FESEM analysis. The willemite NPs aggregate from the smaller to larger particles with an increase in calcination holding time from 1–4 h with the sizes ranging between 19.74–29.71 nm. The energy values obtained from the experimental band gap decreased with increasing the holding time over the range of 5.39 eV at 1 h to at 5.27 at 4 h. These values match well with band gap obtained from the Mott and Davis model for direct transition. The findings in this study are very promising and can justify the use of these novel materials as a potential candidate for green luminescent optoelectronic applications.

## 1. Introduction

Wide band gap semiconductor nanoparticles (NPs) have attracted substantial interest over the last two decades from researchers and scientists in various fields of human endeavour across the globe due to their abundant physical and chemical attributes [1]. Materials in nano scale like the wide band gap semiconductors NPs exhibit unique properties such as the quantum confinement effect and an increased surface area to volume ratio that distinguish them from those of their bulk counterparts [2]. These semiconductor NPs such as silicates, nitrides and sulphides have been widely used in optoelectronics applications due to their unique properties coupled with their natural state [1,3]. Zinc silicate (Zn_2_SiO_4_) or willemite belongs to the functional inorganic wide band gap semiconductors. The compound occurs naturally with minor ores of zinc minerals with a phenacite structure where Zn–O tetrahedral and Si–O tetrahedral share corners, forming hollow tubes that appear parallel to [0001] plane [3,4]. Willemite has potential for green phosphors and laser crystal applications [5,6,7,8,9]. Some other important applications of willemite include the use as adsorbents [10,11], optoelectronics [12,13], as well as in photonic devices [14,15]. 

However, several techniques were reported for the synthesis of willemite by numerous authors. This is including the conventional sol–gel technique [16,17,18] and solid state method [19,20,21,22]. In addition, other researchers reported the production of willemite by hydrothermal method [23,24], co–precipitation method [25], sonochemical method [26], spray drying method [27], spray pyrolysis method [28,29], super critical water methods [30,31] and solvothermal method [32]. It was inferred that the sol–gel technique is one the famous methods for producing semiconductor nanoparticles. In this light, Rasdi et al. produced willemite nanophosphors at sintering temperature of 1000 °C for 2 h prepared using the sol–gel method and obtain an average grain size of 181 nm [33]. In the same vein, Babu et al. fabricated the same material with an average particle size of 100 nm. This involved a lengthy reaction period by allowing the precursor to get evaporated naturally in the air for two or three months and later calcined at 950 °C for 2 h [34]. The solid state method when compared to other techniques like the sol gel often employs higher calcination temperature ranging between 1000–1500 °C [12,20,21]. This technique was reported by Diao et al. [35] where willemite NPs were produced at a temperature between 900–1300 °C and obtained particle size greater than 200 nm. Similarly, the particle size of the willemite produced by Al–nidawi et al. [36] were at the range between 500–1000 nm, and this involved high temperature up to 1400 °C. The willemite NPs synthesized by hydrothermal methods generally involves low calcination temperature between 200–700 °C [37,38,39]. For instance, Zeng et al. [37] synthesized non–uniformly distributed particles of willemite sized between 200–300 nm at the sintering conditions of 230 °C for 12 h. The same procedure was used by Xu et al. [40]. The authors fabricated willemite nanorods with the width and length of about 0.25 × 1 μm by mixing the solvent of ethidene diamine and water at 200 °C for six days. 

Nevertheless, most of these methods are faced with challenges in their application due to the high heat treatment conditions involved, longer reaction period for several hours, complicated procedures, and toxic chemical pollution released to the immediate environment. These problems can be curtailed using a polymer thermal treatment method. This technique has become a vital tool in the synthesis chemistry to produce a novel nano–sized metal oxide semiconductor materials at lower cost in recent years [41,42,43]. Based on the authors’ best knowledge, there are not yet reports on the synthesis of willemite nanoparticles using a polymer thermal treatment method. Therefore, this study presents an optimized production of willemite NPs at 900 °C for several holding times between 1–4 h. Apart from that, the effects of the calcination holding time on the microstructure and optical properties of the willemite NPs have also been extensively studied and discussed in detail.

## 2. Results and Discussion

### 2.1. Thermogravimetric Analysis

**Figure 1:**
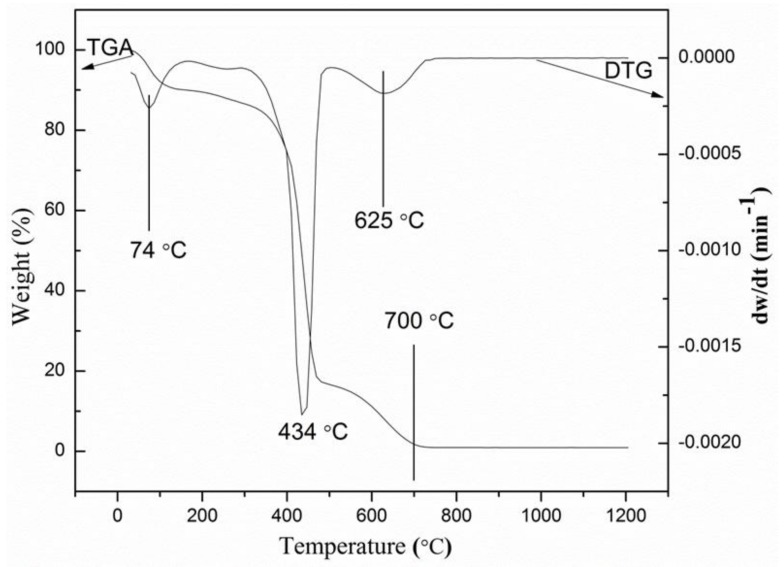
Thermogravimetric (TG) and thermogravimetric derivative (DTG) curves for PVP at a heating rate of 10 °C/min.

The thermogravimetric analysis and differential thermal analysis (TGA–DTA) of the uncalcined samples (metallic salt combined with PVP) offered significant information on the appropriate temperature to decompose PVP and other unwanted anions to obtain willemite NPs. Figure 1 illustrates the weight loss percentage of the uncalcined sample versus the temperature. The thermogram exhibits four distinct decomposition curves. The initial weight loss recorded is observed to be at 74 °C (8%), which is assumed to be caused by the moisture content entrapped in the sample [41]. The second weight loss detected at approximately 205 °C is ascribed to volatile components such as peroxide residue [42,44]. The third weight loss forms the peak observed at 434 °C, which implies that significant amounts (78%) of the C–C in the polymeric chain have been decomposed. The fourth curve on the thermogram is the decomposition due to the remnant of ester group from the PVP observed at 625 °C. The thermogram reveals no further weight loss as the peak of the temperature reaches 700 °C; this can be attributed to the entire decomposition of PVP, thereby turning it into a carbonaceous product leaving behind a residue of willemite NPs. Thus, the thermogram reveals that, for the fabrication of willemite NPs to be achieved, the optimum calcination temperature must be maintained beyond 700 °C. This is in good agreement with the work reported by other authors who claimed that the formation temperature of the willemite phase is between 700 and 800 °C [16,26]. 

### 2.2. Raman Analysis 

**Figure 2:**
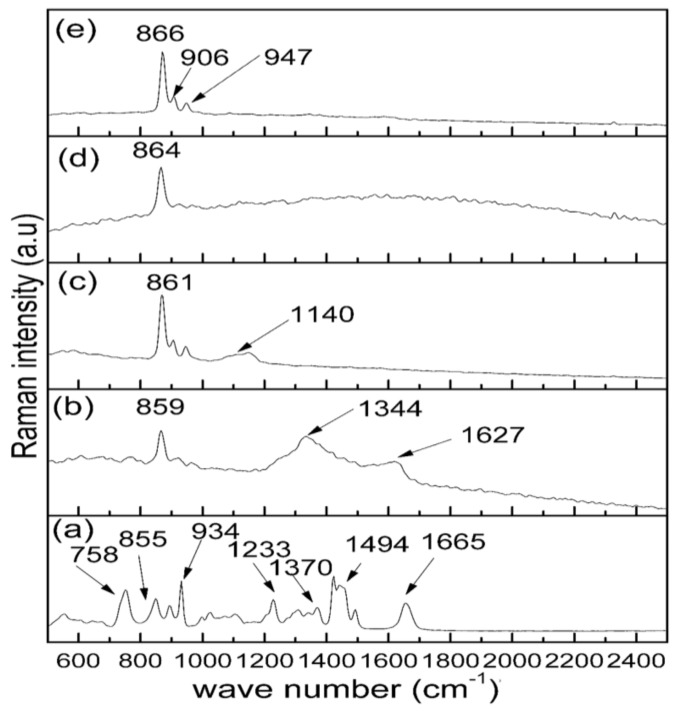
Raman spectra of (**a**) uncalcined sample and willemite NPs calcined at 900 °C over a range of calcination holding times of (**b**) 1 h, (**c**) 2 h, (**d**) 3 h and (**e**) 4 h.

Raman spectroscopy was employed in this work to study the organic and the inorganic properties of the samples before and after the calcination process as shown in Figure 2. The uncalcined sample (Figure 2a) at room temperature demonstrates an organic behaviour due to the appearance of several vibrational bands related to PVP. Table 1 presents the Raman vibrational bands and their corresponding assignments. The absorption peak positioned at 758 cm^–1^ is related to the C–C ring vibration and the one at 934 cm^–1^ is for the C–C ring breathing [45]. The peaks appear at 1233 and 1370 cm^–1^ due to the C–C back bone and CH deformation, respectively [45,46]. The peak observed at 1665 cm^–1^ is related to C=O, while the band at 1494 cm^–1^ is ascribed to the CH_2_ scissors vibration [45]. 

The samples later underwent a calcination process at a constant temperature of 900 °C for several holding times ranging from 1–4 h as shown in Figure 2b–e. The formation of crystalline willemite phase was obtained after the calcination process. The samples produced at the holding time of 1 h and 2 h exhibit a sharp peak attributed to the willemite phase and some peaks ascribed to the remnants of the organic sources (from PVP) positioned at 1344, 1627 and 1140 cm^–1^, respectively. There are no traces of PVP after the samples are calcined at the holding time of 3 and 4 h, respectively. Thus, the spectra of the crystalline willemite NPs reveal vibrational peaks fixed at 866, 906 and 947 cm^–1^ initiated from a surface group of siloxane [47,48]. The small shifting to the lower wavenumber of the most intense Raman bands is due to the enhancement in the crystal lattice of the material produced. The most strong peak at 866 cm^–1^ is ascribed to the willemite NPs crystalline vibrational band [49].

### 2.3. FT–IR Analysis

**Figure 3:**
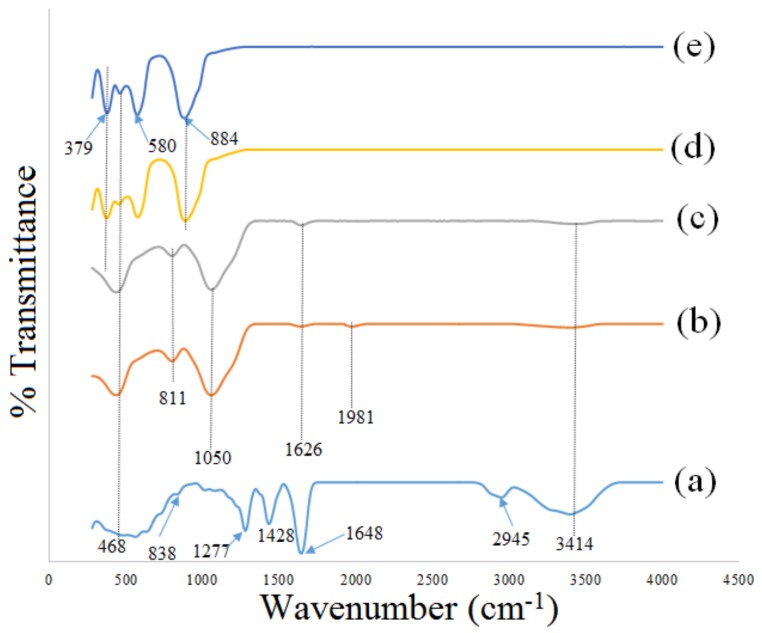
FT–IR spectra of (**a**) uncalcined sample and willemite NPs calcined at 900 °C over a range of calcination holding times of (**b**) 1 h, (**c**) 2 h, (**d**) 3 h and (**e**) 4 h.

FT–IR spectroscopy is used in analyzing multi–functional groups and obtain the related information concerning the sample phase composition before and after the calcination process. To investigate the interactions of the chemical component and phase composition of metallic salts and PVP, Figure 3a–e illustrates the FT–IR spectrum of the samples before and after calcination process. The uncalcined sample at room temperature presented in Figure 3a exhibits several absorption peaks at bands of 3414, 2945, 1648, 1428, 1278, 850, and 639 cm^–1^ related to the presence of organic source from PVP [41,42,50,51,52]. For more comprehensive details on the FT–IR bands and their assignment, a tabular presentation is provided in Table 2. Based on Figure 3b–e, the samples are calcined at 900 °C for a period of 1–4 h; this causes the decomposition of the organic source from PVP as depicted in Figure 3d,e. The exception is for the willemite NPs produced at 1 and 2 h calcination holding time respectively, where the remnants of PVP were still present. The absorption bands sighted at a wavenumber of 1626, and 3414 cm^–1^ are ascribed to C=O and C–H bending vibrations from the methylene group [42]. The peaks at the range between 350–580 cm^–1^ are related to ZnO_4_ symmetric and asymmetric stretching vibrations [26]. The vibrational bands ranging from 800–1050 cm^–1^ are assigned to the vibrations of the SiO_4_ group [26].

### 2.4. X-ray Diffraction Analysis

**Figure 4:**
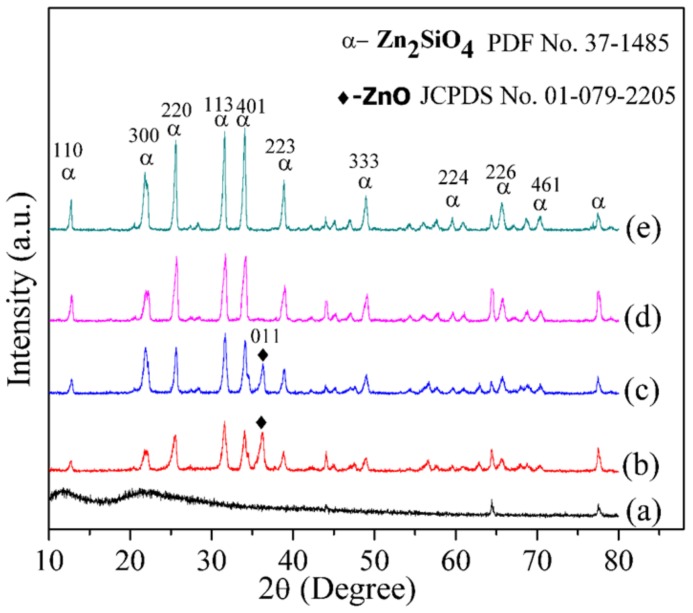
X-ray diffraction (XRD) of (**a**) uncalcined sample and willemite NPs calcined at 900 °C over a range of calcination holding times of (**b**) 1 h, (**c**) 2 h, (**d**) 3 h and (**e**) 4 h.

**Figure 5:**
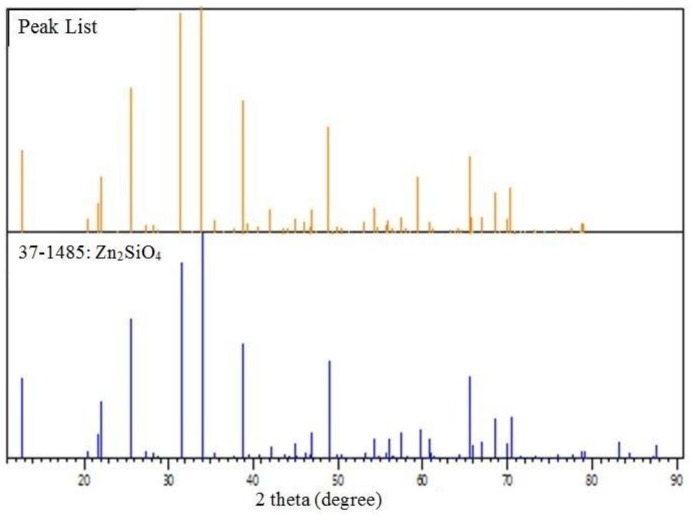
XRD reference patterns of willemite NPs produced at the optimum synthesis condition of 900 °C for 3 h.

The impact of the calcination holding time on the fabricated willemite NPs was studied using the diffraction peaks generated by the XRD. Prior to the heat treatment process, the uncalcined sample at room temperature was analysed as shown in Figure 4a. The broad spectrum observed indicates that the sample is amorphous at room temperature (30 °C) as there are no clear peaks that suggest the crystalline phase of willemite. This is in agreement with our findings elsewhere when ZnO precursor is embedded with PVP [43]. However, upon the calcination at 900 °C for 1 and 2 h holding time respectively, shown in Figure 4b,c, the spectrum pattern exhibits sharper and narrower peaks. Although there is no complete formation of the willemite phase due to the appearance of a trace of the unreacted phase of ZnO at the diffraction peak position of 2θ = 36.40° which corresponds to the 001 plane [53]. The calcination holding time is increased to 3 and 4 h respectively, and this enables the unreacted ZnO atoms to diffuse towards the silica matrix and induces the formation of a single crystalline phase of α–willemite. The diffraction peaks are indexed to the standard pattern of α–willemite (JCPDS No 37-1485), with a space group of R–3, and cell constant a = b = 13.947 Å, c = 9.3124 Å [26]. The crystallinity of the willemite NPs is higher with an increase in the calcination holding time. The phenomenon of crystalline size growth with increasing calcination holding time is stemmed from the enhancement of the crystalline volume to the surface ratio, which occurs due to particle size expansion [44]. The XRD result is in good agreement with the Raman and FT–IR spectra shown in Figure 2 and Figure 3, respectively. For this reason, the calcination holding of 900 °C 3 h is endorsed as the optimum condition for synthesizing willemite NPs with minimum energy consumption. The diffraction standard reference pattern and the peak list of the optimum synthesis condition are presented in Figure 5. The mean crystalline size is calculated from Scherer’s formula shown in Equation (1). The size is between 18.23–27.40 nm and it increases with the increase in the calcination holding time. This is in good conformity with particle size values shown in Table 3. 

### 2.5. HR-TEM Analysis

**Figure 6:**
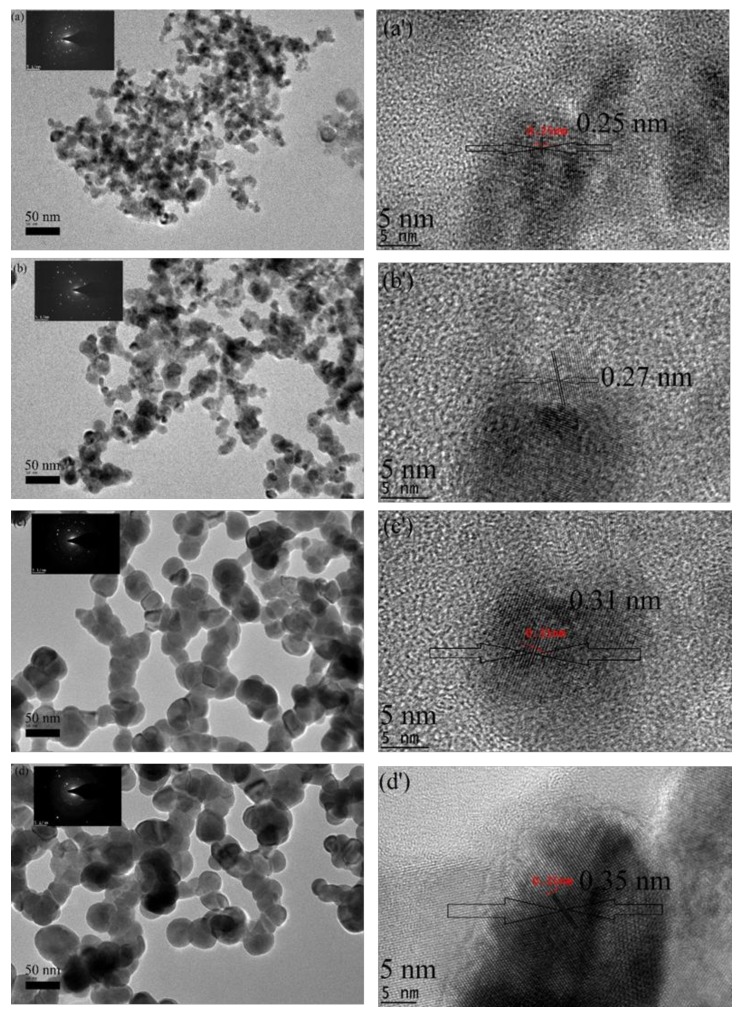
HR-TEM images and the corresponding lattice measurement for willemite NPs produced at 900 °C prepared at different calcination holding times: (**a**,**a'**) 1 h; (**b**,**b'**) 2 h ; (**c**,**c'**) 3 h; and (**d**,**d'**) 4 h.

**Figure 7:**
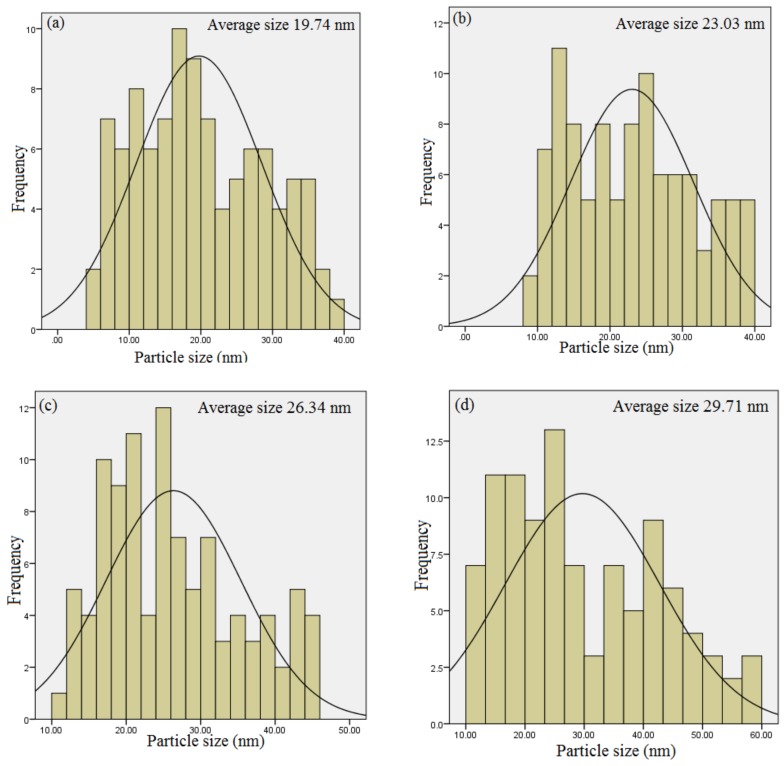
Particle size distribution of willemite NPs calcined at 900 °C for several holding times (**a**) 1 h, (**b**) 2 h, (**c**) 3 h and (**d**) 4 h.

The effect of the calcination holding time on the microstructure and particle size distribution of the willemite NPs is presented by the HR-TEM images and selected area electron diffraction (SAED) pattern shown in Figure 6. The material exhibits uniformity with relatively homogeneous size distribution. The willemite NPs aggregate from the smaller to larger particles with an increase in calcination holding time from 1–4 h. This is associated with the fact that a higher heating process induces faster nucleation growth of the nanoparticles thereby increases the chances of agglomeration [33,44,50]. The average particle size of the material presented in Table 3 and Figure 7 are estimated to be in the range between 19.74 to 29.1 nm using ImageJ software (version 1.40g, U.S. National Institutes of Health, Bethesda, MD, USA). In the HR-TEM images, the distance between the two adjacent planes (lattice distance), d, is measured to be lesser at a lower calcination holding period, and the lattice increases with a corresponding increase in the holding time. The SAED was similarly conducted to confirm the polycrystalline nanostructure of the material at different calcination holding times. The results obtained are in good conformity with the outcomes from the XRD analysis and correlates to those reported by previous researchers [26,54]. In this paper, a simple polymer synthesis technique was optimized to produce uniform and fine willemite NPs at the calcination temperature of 900 for 3 h. Comparison to other synthesis techniques reported on similar material is shown in Table 4. It is fascinating to note that this technique is more promising in terms of low production cost, simplicity in the process, better control of the shape and particle size, shorter reaction period, low calcination temperature, and lack of harmful chemicals released to the environment.

### 2.6. FESEM-EDX Analysis

**Figure 8:**
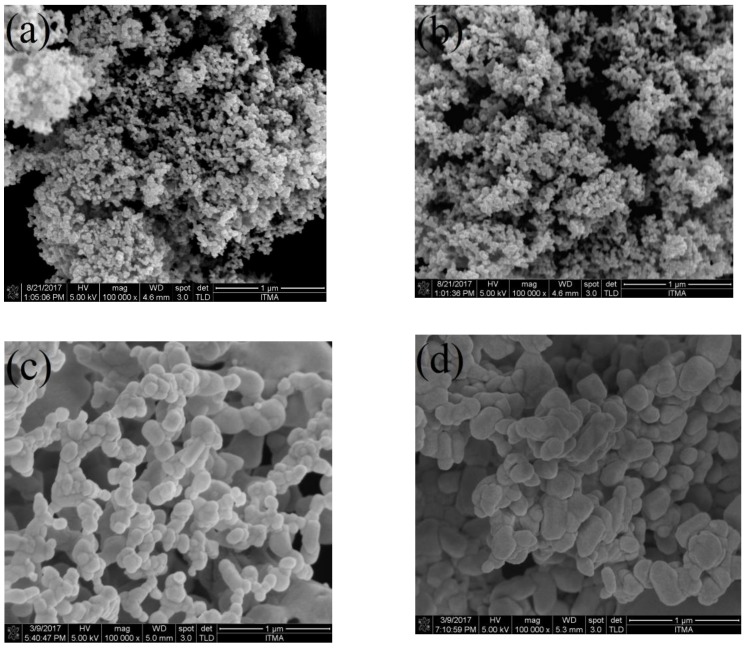
FESEM images for willemite NPs produced at 900 °C with different calcination holding times: (**a**) 1 h, (**b**) 2 h, and (**c**) 3 h and (**d**) 4 h.

The surface morphology properties, particle dispersal and elemental composition of the synthesized willemite NPs were studied using field emission electron microscopy (FESEM) equipped with energy-dispersive X-ray spectroscopy (EDX). The images were viewed at the operating voltage of 5 kV. The images in Figure 8 illustrate how the calcination holding time affects the surface morphology properties. The willemite NPs produced with lower calcination holding time of 1 and 2 h, respectively, exhibit a tiny particle—like microstructure, and non–homogeneity in the distribution. Nevertheless, with the increasing calcination holding time to 3 and 4 h respectively, (Figure 8c,d), the samples had shown NPs of different sizes with strong necking between the particles. Consequently, it is fascinating to note that the process of calcination at higher holding times had affected the surface morphology and NP arrangements. This is due to the Ostwald ripening growth mechanism, where larger NPs grow at the expense tiny particles [56]. The dimension of willemite NPs is increased with increasing the calcination holding time [42,50]. 

**Figure 9:**
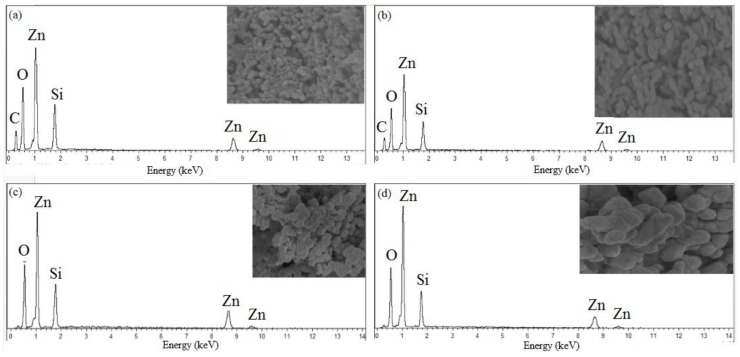
EDX spectrum of willemite NPs calcined at 900 °C with different calcination holding times: (**a**) 1 h, (**b**) 2 h, (**c**) 3 h and (**d**) 4 h.

The purity and elemental composition of willemite NPs formed were investigated using the EDX analytical technique. The fundamental working principle of this technique is such that each distinct element possesses a unique atomic structure allowing a unique set of peaks on its X-ray spectrum. The spectrum in Figure 9 presents the EDX of the willemite NPs calcined at 900 °C for different heat treatment conditions between 1–4 h. The corresponding peaks of Zn, Si and O detected in the spectrum confirm the purity of the willemite NPs formed, and there are no loss of elements in the process [26]. 

### 2.7. UV–Vis Analysis

**Figure 10:**
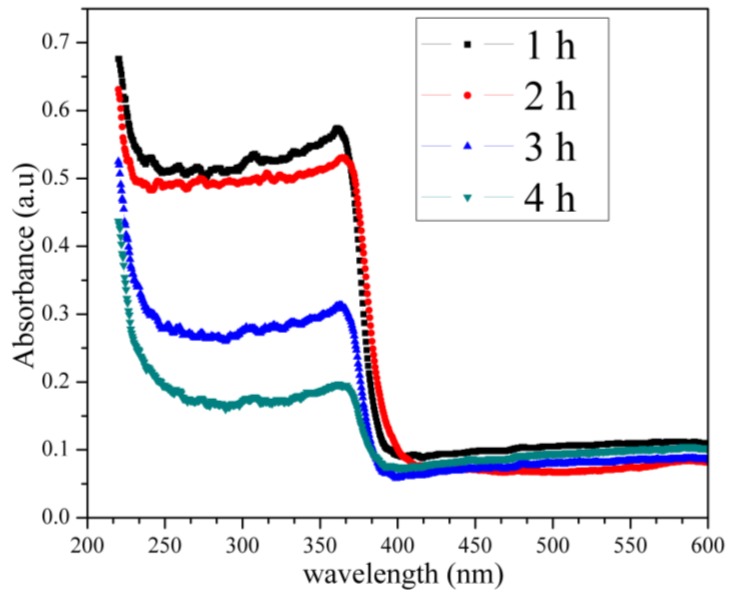
The optical absorbance spectra of the willemite NPs fabricated 900 °C for several holding time.

The light absorption properties of the synthesized willemite NPs are presented in Figure 10. It is observed that the materials offer a maximum absorption peak at a wavelength of 375 nm for the samples calcined at 900 °C for 1 and 2 h, respectively, which can be ascribed to the transition of election from the valence band to the conduction band. The absorption peak observed at 260 nm is due to the non-bridging oxygen hole centres [57]. The absorption edge, as well as the intensity of the spectrum, suffers a decrease toward the UV region due to particle size increase for samples calcined at the 900 °C for 3 and 4 h holding times, respectively [58]. The shift to the lower wavelength of 369 nm can be attributed to the relative deterioration of the crystal quality of ZnO, which allows for the formation of a single phase of willemite NPs as evidenced by the XRD result [53].

**Figure 11:**
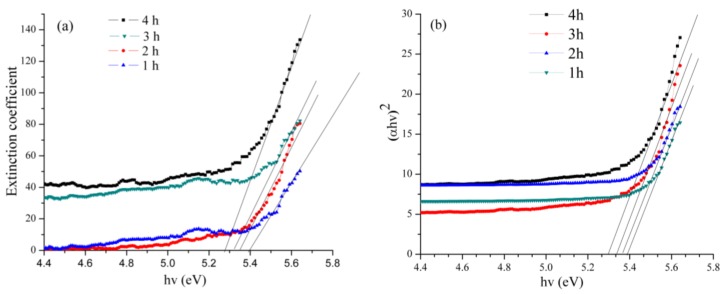
Extraction of: (**a**) the experimental optical band gap using extinction coefficient and (**b**) optical band gap from Mott and Davis Model for *n* = 1/2 transition.

The optical absorbance spectra of the willemite NPs produced at 900 °C with different calcination holding time. The influence of the calcination holding time is observed on the optical band gap of the entire samples as illustrated in Figure 11. Our findings revealed that the $E_{g}$ values obtained from the experimental band gap using Equation (2) decreased with increasing the holding time over the range of 5.39 eV at 1 h to 5.27 at 4 h. It has been reported that the electronic performance of a semiconductor material depends heavily on the synthesis condition such as heat treatment in which the calcination temperature affects the crystallinity of the material [59]. In this light, when the particle size of a certain material is reduced, the number of atoms that made up the particle reduces as well. This renders the valence and conduction electrons less attractive to the ion core of the particles and thereby causes an enlargement in the band gap [60]. The experimental band gap obtained matches well with the Mott and Davis model for *n* = 1/2 shown in Equation (3). Thus, based on the comparison with Mott and Davis model, it can be understood that, for the willemite NPs, the band gap energy arises due to the direct allowed transition (*n* = 1/2). Hence, this is in good conformity with the blue shift in the UV spectra of the product reported in this work. Table 5 illustrates the variation of band gap energy for willemite NPs for different values of *n* transition. The wide band gap values obtained in this study are in agreement with those reported by other researchers [36,53,61,62,63].

### 2.8. Photoluminescence Analysis

**Figure 12:**
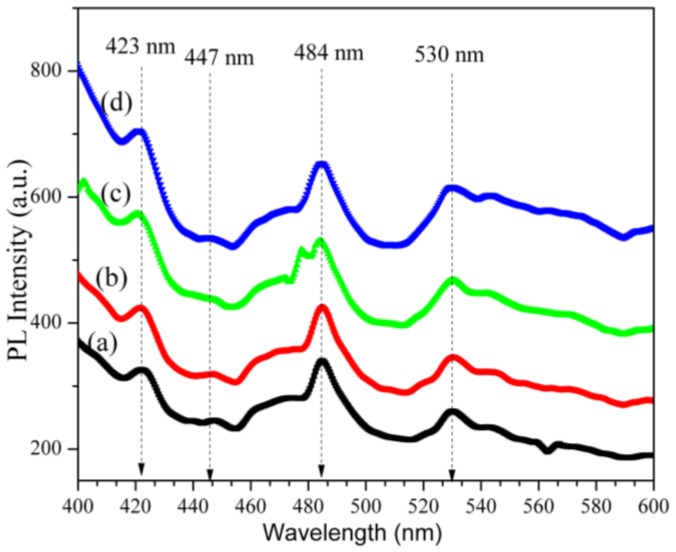
PL spectra of the willemite NPs calcined at 900 °C with different calcination holding times: (**a**) 1 h (**b**) 2 h (**c**) 3 h and (**d**) 4 h.

The material’s structural internal energy levels were studied using a PL spectrometer (Perkin Elmer LS 55, Waltham, MA, USA). The analysis was used to estimate the extent of luminescence, with the peaks revealing the measure of the internal energy levels of the fabricated willemite NPs. To determine the effects of calcination within the range of 1–4 h was considered. In this light, Figure 12 presents the emission properties of the samples under the excitation at a 350 nm wavelength conducted in a normal ambient condition. The green emission sighted at 530 is attributed to the obvious transition of the electrons between the valence and conduction. It was reported that the oxygen defects can be attributed to the crystallization process induced by nanocrystals when the samples were being heat treated [64,65]. This led to a change in the structural arrangement [64]. In this light, the emission peak at 420 and 447 is attributed to an oxygen defect in the blue region, and it is often called the blue emission [53]. The emission peak at 485 is anticipated to be due to zinc interstitial [66,67]. As can be comprehended in the emission spectra, the intensity of the spectrum is altered by the rise in the calcination holding time for the entire sample. Higher emission intensity often occurs due to the enhanced crystallinity [68]. The heat treatment process is often considered an active means for enhancing the emission intensity of phosphor material because of its low surface defects and a higher degree of crystallinity [69,70]. Whenever a surface area is reduced, the material crystallinity increases as well as the PL intensity increases [71]. According to Fu et al. [72], there are certain factors responsible for the heightening in emission intensity in ZnO loaded SiO_2_ matrix system; these include the surface passivation of ZnO, interface state formed between ZnO and SiO_2_ and the excitation process in the SiO_2_.

## 3. Material and Methods

Polyvinyl pyrrolidone (PVP) 2900 molecular weight was purchased from Sigma Aldrich (St. Louis, MO, USA) and used as a stabilizer to stabilize the NPs and as a capping agent to reduce agglomeration. Metallic salt of zinc acetate dihydrate reagent, (Zn(CH_3_COO)_2_.2H_2_O) (M_w_ = 219.49 g/mol)) and silicon tetraacetate reagent (Si(OCOCH_3_)_4_) (M_w_ = 264.26 g/mol) with over 99% purity (from Sigma Aldrich) were used as sources of the metallic precursor. Deionized water with a resistivity and conductivity of 18.2 MΩcm^–1^ and 0.055 μScm^–1^ was used to dissolve the chemicals. All of the chemicals were used as purchased and so no further purification was carried out. An aqueous solution of PVP was prepared by dissolving 0.04 g/mL of PVP in 100 mL of the deionized water. The solution was continuously stirred using a magnetic stirrer for 2 h until all the precipitation disappeared. Equal concentrations (0.01 mmol each) of metallic salts of Zn(CH_3_COO)_2_.2H_2_O and Si(OCOCH_3_)_4_ were added to the polymer solution and stirred continuously for another 2 h. The solution was then transferred into a glass evaporation dish and placed into an electric oven at 80 °C for a day. The resulting yellowish solid obtained was crushed to a powder form using a mortar and pestle. The powder was subjected to a calcination process in a box furnace at a constant temperature of 900 °C for several holding times between 1–4 h for the crystallization and formation of the willemite NPs.

### Characterization

In this study, several techniques were used to study the effects of several calcination holding time on the properties of the willemite NPs calcined at 900 °C. Thermogravimetric analysis (TGA) and its first derivative (DTG) were conducted using a TGA/DSC 1HT model, (METTLER TOLEDO, Shah Alam, Selangor, Malaysia). The decomposition properties of the sample (metallic precursor embedded with PVP) were measured under natural conditions within a temperature range between 30 and 1000 °C. The structural properties and phase of the crystalline sample were analysed using an X-ray diffraction spectrometer (XRD Shimadzu model 6000 Lelyweg1, Almelo, The Netherlands) using Cu kα (0.154 nm) as a radiation source to generate diffraction peaks from the sample within a 2θ angle range of 10–80°. Scherer’s formula is applied to calculate the crystalline size of the willemite NPs for the most intense peak, expressed in Equation (1) below:

(1) D=0.9λβcosθ

where *D* is the crystalline size measured in (nm), *θ* is the Bragg’s angle and *λ* is the X-ray wavelength of Cukα (0.154 nm), while *β* is the full width of the diffraction at half of the maximum intensity measured in radians. The bond formation and functional groups of the samples before and after the calcination process were studied using infrared spectroscopy (FT–IR, Perkin Elmer model 1650, Labexchange, Swabian Burladingen, Germany). The chemical bond and shift where studied using Raman spectroscopy. The analysis of the samples before and after the calcination process was conducted using a ‘Wissenschaftliche Instrumente und Technologie’ (WITec) Raman spectrometer, Alpha 300R (WITec GmbH, Ulm, Germany). The Raman frequency was acquired with a laser excitation wavelength of 532 nm and an integration time of 5.03645 (s). The particle size and distribution were viewed using high resolution transmission electron microscopy (JEOL HR–TEM model 3010, Tokyo, Japan) with an accelerating voltage of 200 kV. The surface morphology of willemite NPs was viewed using field electron scanning microscopy (FESEM) with an accelerating voltage of 5 kV equipped with EDX using an FEI Nova NanoSEM 230 (FEI, Hillsboro, OR, USA). The optical absorption properties were probed using the UV–Vis spectrometer (Shimadzu model UV–3600, Kyoto, Japan). Based on the absorbance spectra from the UV–Vis spectrometer, the experimental optical band gap could be evaluated using the relationship between absorption and extinction coefficient, which is given by the equation as follows:

(2) $$k\;=\frac{\alpha \lambda }{4\pi },$$

where *k* is the extinction coefficient. The values obtained from the experimental bang gap are compared with the energy gap ($E_{g}$) obtained using the Mott and Davis equation [73]. Assuming a transition between valence and conduction bands, the relation is given by the function as follows:

(3) $${\left(\propto hv\right)}^{1/n}=A\left(hv-E_{g}\right),$$

where A is a constant, and hv is the photon energy while the optical energy band is denoted by $E_{g}$. As shown in Figure 11b and Table 4, the characteristics ${\left(\propto hv\right)}^{1/n}$ are plotted against the hv values, where the optical band gap is obtained by extrapolating the linear axis to where ${\left(\propto hv\right)}^{1/n}$ is equal to zero. The value of *n* is depending on the kind of the transition; whether direct transitions (where the *n* value is given as 1/2 or 3/2) or indirect transitions, (where *n* is given as 2 or 3) and whether the transition is allowed or forbidden. Photoluminescence (PL) (Perkin Elmer LS 55, Waltham, MA, USA) was used to analyse the optical properties of the materials at room temperatures, within the wavelength of 200–800 nm.

## 4. Conclusions

The influence of the heat treatment condition on the microstructural and optical properties of willemite NPs was investigated. The Raman, FT–IR and XRD spectra of the samples confirmed the crystallization and formation of willemite NPs after the calcination process. The calcination holding time of 3 h was adopted from these results as the optimum condition for producing willemite NPs with minimum energy consumption. The XRD analysis showed the formation of a willemite phase and the presence of the unreacted ZnO phase for the samples produced at 900 °C for 1 and 2 h. Further increase in the calcination holding time for 3 and 4 h had respectively revealed the formation of a pure willemite phase. The crystalline size of the material increases alongside with the increase in the calcination holding time ranging from 18.23–27.40 nm. From the HR–TEM images, the lattice distance “d” between the two adjacent planes was found to be lesser at a lower calcination period, and the lattice increases with an analogous increase in the calcination holding time. Similarly, the SAED confirmed the polycrystalline nanostructure of the willemite NPs at different calcination holding times. The light absorption properties of the material offered a maximum absorption peak at a wavelength of 375 nm for the sample calcined at 900 °C for 1 h and 2 h and shifted to a lower wavelength of 369 nm at a higher calcination holding time. This was attributed to the relative deterioration of the crystal quality of ZnO, which allowed for the formation of a single phase of willemite NPs. The $E_{g}$ values obtained decreased with the increased holding times over the range 5.39 eV at 1 h to at 5.27 4 h. This was in good agreement with the blue shift in the UV spectra. The PL emission bands revealed a strong blue emission at 485 due to zinc interstitial and the intensity afflicted by the rise in the calcination holding. The results obtained in this study justify that willemite NPs produced had novel potentials as a candidate in green luminescent optoelectronic applications.

## Figures and Tables

**Table 1:** Summary of the Raman absorption features and their assignments.

Wave Number (cm^–1^)	Assignment of Vibration Mode	Reference
800–1100	Stretching vibration of SiO_4_ group	[47,48]
859–866	Crystalline Zn_2_SiO_4_ vibration peak	[48,49]
758	Ring C–C vibration	[45]
885	C–C stretching vibration	[45]
934	C–C Ring breathing	[45,46]
1233	C–C Back bone	[45]
1370	C–H Deformation	[45,46]
1494	CH_2_ Scissors	[45]
1665	C=O (amide)	[45]

**Table 2:** Summary of the FT–IR absorption features and their assignments.

Wave Number (cm^–1^)	Assignment of Vibration Mode	Reference
379–441	Zn–O asymmetric stretching vibration	[26]
580	Zn–O symmetric stretching vibration	[26]
884–884	Si–O stretching vibration	[26]
639–641	C–N=O bending vibration	[52]
838	C–C ring vibration	[51,52]
1277	C–N stretching vibration	[41,50,51]
1402–1429	–C–H– bending vibration of methylene group	[42]
1648	N–H stretching vibration	[50]
2945	C–H vibration	[41,50,51]
3414	N–H bending vibration	[41,50,51]

**Table 3:** Summary of the structural and optical features of the synthesized willemite NPs calcined at 900 °C with different heat–treatment condition.

Calcination Holding Time (h)	Peak Position (2θ)	FWHM	D_XRD_ (nm)	D_TEM_ (nm)	Experimental E_g_ (eV)
**1**	33.86	0.476	18.23	19.74	5.39 ± 0.04
**2**	33.90	0.388	21.70	23.03	5.35 ± 0.04
**3**	33.96	0.351	22.29	26.34	5.34 ± 0.04
**4**	33.98	0.308	27.40	29.71	5.27 ± 0.04

**Table 4:** Characterization comparison of the produced willemite NPs obtained other similar work.

Method	Precursors	Synthesis Conditions	Particle Size (nm)	Morphology	References
Solid–state	Zinc oxide, silicon oxide powder	calcination: 1330 °C, 4 h	1000–5000	Larger grain size	[22]
Hydrothermal	zinc nitrate, silicon oxide	700 °C, 10 h	300–1000	Non–uniform distribution	[39]
Super critical water	zinc hydroxide, Amorphous silica	30 MPa, 1 h, 400 °C	1000–2000	Non–uniform nanorods, distribution	[55]
Sol–gel method	Tetraethylorthosilicate, and zinc acetate dihydrate	1200 °C, 2 h,	70–80	Non–uniform particle distribution	[18]
Spray pyrolysis	zinc acetate, tetraethyl orthosilicate, sulpuric acid	1000 °C, 10 h	200–1000	Spherical particles, aggregate morphology	[29]
Sonochemical	Tetraethylorthosilicate, and zinc acetate dihydrate	950, 2h	50	Uniform distribution	[26]
Polymer thermal treatment	zinc acetate dihydrate Silicon tetraacetate	900 °C 3 h	26	Uniform Nanoparticle	This Paper

**Table 5:** Variation of band gap energy for willemite NPs calcined at 900 °C for different calcination holding times.

Optical Bandmgap (eV)	*E*_g_ (Experimental)	Direct Allowed Transition *n* = 1/2	Direct Forbidden Transition *n* = 3/2	Indirect Allowed Transition *n* = 2	Indirect Forbidden Transition *n* = 3
**1 h**	5.39 ± 0.04	5.38 ± 0.04	5.32 ± 0.04	5.23 ± 0.04	5.43 ± 0.04
**2 h**	5.36 ± 0.04	5.35 ± 0.04	5.27 ± 0.04	5.17 ± 0.04	5.38 ± 0.04
**3 h**	5.34 ± 0.04	5.33 ± 0.04	5.21 ± 0.04	5.07 ± 0.04	5.27 ± 0.04
**4 h**	5.27 ± 0.04	5.25 ± 0.04	5.16 ± 0.04	4.72 ± 0.04	5.10 ± 0.04
